# The possible association of clusterin fucosylation changes with male fertility disorders

**DOI:** 10.1038/s41598-021-95288-w

**Published:** 2021-08-02

**Authors:** Ewa Janiszewska, Izabela Kokot, Iwona Gilowska, Ricardo Faundez, Ewa Maria Kratz

**Affiliations:** 1 Department of Laboratory Diagnostics, Division of Laboratory Diagnostics, Faculty of Pharmacy, Wroclaw Medical University, Borowska Street 211A, 50-556 Wrocław, Poland; 2 University of Opole, Institute of Health Sciences, Collegium Salutis Humanae, Katowicka Street 68, 45-060 Opole, Poland; 3 Clinical Center of Gynecology, Obstetrics and Neonatology in Opole, Reference Center for the Diagnosis and Treatment of Infertility, Reymonta Street 8, 45-066 Opole, Poland; 4 InviMed Fertility Clinics, Rakowiecka Street 36, 02-532 Warsaw, Poland

**Keywords:** Molecular biology, Biomarkers, Diseases

## Abstract

In the seminal plasma (n = 118) and serum (n = 90) clusterin (CLU) the fucosylation and the expression of selected fucosyltransferases (FUTs) were analyzed. Samples from infertile men were divided into groups based on the results of the standard semen analysis: normozoospermic (N), teratozoospermic (T), asthenoteratozoospermic (AT) and oligoasthenoteratozoospermic (OAT). The CLU fucosylation was analyzed using lectin-ELISAs with biotinylated lectins specific to α1,3-, α1,2-linked antennary fucose, and α1,6-linked core fucose (LTA, UEA, and LCA, respectively). The concentrations of FUT3 and FUT4, reflecting the expression of Le oligosaccharide structures, were measured using ELISA tests. The differences in serum CLU and FUT4 concentrations, and in the expression of core fucose and antennary fucose α1,2-linked in CLU glycans between the N group and other groups examined suggest that the disturbances in sperm count, motility, and morphology are not the only cause of male infertility. Lack of similarities between levels of examined parameters in blood serum and seminal plasma may suggest the differences in mechanisms leading to glycoproteins glycosylation. It confirmed the observed differences in concentrations of seminal plasma CLU, FUT3, and FUT4 between the OAT group and N, T, AT groups, indicating that decreased sperm count may be related to these parameters expression. The serum CLU concentrations and expression of core fucose and fucose α1,2-linked in CLU, seem to be good markers differentiating normozoospermic men from those with abnormal sperm parameters, which was not observed for seminal plasma.

## Introduction

### Male infertility issue

Male infertility is a growing problem, especially among developed countries^1^. Globally, 20–30% of infertility cases are because of the male factor alone, and another 20–30% due to male and female factors^2^. In the European region, including Poland, it is estimated that nearly 15% of pairs are trying to get pregnant unsuccessfully, and the male factor alone contributes to approximately 50% of cases^3^. Routinely performed semen analysis according to the WHO criteria published in 2010 is often not enough to determine the true cause of male fertility problems. Some pathological values in the ejaculate are only medical hints of male fertility^4^. Moreover, seminal analysis of approximately 15 percent of infertile men result in no pathological values, and these cases are regarded as idiopathic^5,6^. It is worth noting that there is no detailed data from the past decade concerning this issue. Hence, the early specific male infertility biomarkers, including those helpful in idiopathic infertility diagnosis, are still missing. The molecular mechanisms responsible for male fertility problems are still not fully understood.

### Clusterin as one of the glycoproteins present in human semen

Seminogram provides information about the spermatozoa, which makes up only 5% of the human ejaculate. The remaining 95% constitutes seminal plasma, playing an important role in the proper sperm cell production and maturation. This mixture of lipids, inorganic ions, and glycoproteins is responsible for the physiological fertilization process^7–9^. Proteomic studies reported that the seminal plasma of fertile men contains over 6000 proteins, among which the majority belongs to glycoproteins^10^. Milardi et al.^11^ identified 83 proteins present in seminal plasma samples of fertile men, and one of them was clusterin (CLU, also called Apolipoprotein J, ApoJ).

Serum CLU is synthesized in most human cells existing in all body fluids and seminal plasma CLU derived from testis, epididymis and seminal vesicles^12^. Clusterin is one of the main seminal plasma glycoproteins, contains about 30% carbohydrates^13^ and occurs in two main forms: secretory (sCLU, molecular weight ~ 80 kDa) and nuclear (nCLU, molecular weight ~ 55 kDa). The latter form appears in the nucleus due to some cytotoxic events; seminal plasma contains the secretory form of CLU^14,15^. Some authors maintain that clusterin concentration in seminal plasma is several times higher than in serum^12^; however, the literature data concerning this issue is unclear. The human’s sCLU gene is located in chromosome 8, and its structure is well preserved, ordered into nine exons and eight introns. An initial 22 amino acids leader peptide transfers the precursor of sCLU (pre-sCLU) to the endoplasmic reticulum (ER). In subsequent stages, the 60-kDa pre-sCLU undergoes proteolysis, forming α- and β-subunits and is glycosylated, creating a mature disulfide-linked heterodimeric secretory CLU. It is believed that the α subunit has three N-glycosylation sites, and β subunit has four N-glycosylation sites; however, an in vitro observation verified a maximum of six N-glycosylation sites in the whole particle of this protein^16,17^.

Clusterin performs many various functions in the human body, especially in the reproductive system. It is associated with high-density lipoproteins, takes part in lipid transport, participates in cell adhesion and cytoprotection at the tissue–fluid interfaces^18,19^. In the male reproductive tract, clusterin is engaged in the semen liquefaction through interactions with epididymal protease inhibitor (eppin) present on the spermatozoa surface^18^. It is worth noting that sperm oxidation damage is recognized as one of the causes of male infertility, and CLU is a sensitive biomarker of oxidative stress. Clusterin performs chaperone function, which is similar to the function of small heat shock proteins. It is related to the fact that CLU binds unfolded proteins and inhibits their aggregation regardless of the presence of ATP^19^. CLU is also liable for immune tolerance for male antigens in the female reproductive tract. Merlotti et al.^20^ documented that seminal CLU is a novel DC-SIGN (dendritic cell-specific ICAM-3-grabbing nonintegrin) ligand and can perform its role properly only in the highly fucosylated form. They hypothesized that taking into account the fact that female tolerance for male antigens requires *inter alia* DC’s, seminal clusterin may be one of the crucial elements of female tolerance induction^20^.

Protein glycosylation is one of the most common and most important posttranslational modifications affecting many molecular processes which determines protein function^21–23^ since the protein-carbohydrate interaction is responsible for many of cells’ adhesive properties and play a key role in the recognition between the sperm cell and oocyte^24^. Saraswat et al.^25^ carried out a huge N-glycoproteomic study and distinguished fifty glycoproteins, among which the clusterin was one of the most interesting. The authors found four N-glycosylation sites for this glycoprotein and emphasized that CLU had the highest glycan content. The authors proposed 43 various glyco-variants of clusterin; all of them were the complex type with terminal sialic acid or galactose. CLU glycans contained fucose, Le^x^/Le^a^, blood group H or Le^y^/Le^b^ oligosaccharide structures^25^. Considering that fucose is one of the most important glyco-element of seminal clusterin, comprehensive studies concerning CLU fucosylation may contribute to the knowledge of the role of fucosylated glycans in the development of male infertility. Our aim was to check a clusterin fucosylation profile and degree in seminal plamas and blood sera of infertile men using a modified semi-quantitative lectin-ELISA assay. Moreover, we would like to check if there are differences in the profile of fucosylation and fucose expression in clusterin glycoepitopes between groups of examined patients, as well as between seminal plasma and blood serum of infertile men.

## Patient samples

Seminal plasma and serum samples were collected from infertile male patients attending the Clinical Center of Gynecology, Obstetrics, and Neonatology in Opole (Poland) and Fertility Clinics InviMed in Warsaw (Poland). Each patient gave informed consent for this study. The study was conducted according to the guidelines of the 2nd Declaration of Helsinki and approved by the Bioethics Committee of Wroclaw Medical University (No. KB 549/2019 and No. KB 707/2019).

The ejaculates were collected by masturbation into sterile containers after 3–5 days of sexual abstinence. After liquefaction (maximum 60 min at 37 °C), standard semen analysis was carried out using computer-assisted sperm analysis (total number of sperm in ejaculate, sperm concentration, total motility, progressive motility, and morphology), SCA Motility and Concentration, software version 6.5.0.5. (Microptic SL, Barcelona, Spain) and manually (semen volume, pH and viability). All input data in this method were consistent with current WHO semen analysis criteria. Ejaculates were centrifuged at 3500×*g* for 10 min at room temperature, aliquoted, and frozen at − 86 °C until use. Serum samples were obtained by peripheral blood collection and after coagulation centrifuged at 2000×*g* for 10 min at room temperature. Serum aliquots were also stored at − 86 °C until examination. None of the serum samples were haemolysed.

Based on the standard semen analysis (sperm concentration, progressive motility, morphology of spermatozoa), seminal samples (n = 118) were divided into groups: asthenoteratozoospermic (AT, n = 22; ≥ 32% of sperm had abnormal progression and lower than 4% of spermatozoa had normal morphology), oligoasthenoteratozoospermic (OAT, n = 29; sperm count lower than 15 × 10^6^ mL^–1^, ≥ 32% of sperm had abnormal progression and lower than 4% of spermatozoa had normal morphology), teratozoospermic (T, n = 38; lower than 4% of spermatozoa had normal morphology) and normozoospermic (N, n = 29, normal values of ejaculate parameters). Corresponding blood serum samples (n = 90) were divided into asthenoteratozoospermic (AT, n = 16), oligoasthenoteratozoospermic (OAT, n = 27), teratozoospermic (T, n = 31) and normozoospermic (N, n = 16) groups. In the normozoospermic ejaculates, the concentration of spermatozoa was higher than 15 × 10^6^ mL^–1^, and > 4% of sperm exhibited normal morphology, with total motility of ≥ 40% or progressive motility ≥ 32% (0.5 h after ejaculation). None of the seminal samples were leukospermic.

## Methods

### Clusterin concentration

Seminal plasma and blood serum clusterin concentrations were determined using Human Clusterin ELISA kit from Bioassay Technology Laboratory (catalog No. E1189Hu; Shanghai, China) and Human Clusterin Elisa Kit from Invitrogen (ThermoFisher Scientific, catalog No. EHCLU; Frederick, USA), respectively, according to the manufacturer instructions, without any modifications. The coefficients of variations (CV%) for both tests were defined by the manufacturers. The intra-assay CV% for seminal plasma CLU ELISA kit was defined as < 8% and inter-assay CV% was ranged by < 10%. The intra-assay CV% for blood serum CLU ELISA kit was ranged by < 10% and inter-assay CV% was defined as < 12%.

### Fucosyltransferases concentration

FUT-3 concentration was measured using Human Galactoside 3(4)-L-Fucosyltransferase ELISA Kit from Bioassay Technology Laboratory (catalog No. E4361Hu; Shanghai, China), and FUT-4 concentration was measured using Human Fucosyltransferase 4 ELISA kit from Bioassay Technology Laboratory (catalog No. E4612Hu; Shanghai, China) according to the manufacturer instructions, without any modifications. The coefficients of variations (CV%) for both tests were defined by the manufacturers. The intra-assay coefficients of variations were defined as < 8% and inter-assay coefficients of variations ranged by < 10% for both tests. Levels of these enzymes correspond to the expression of Le^a/x/b/y^/sLe^a/x^ and Le^x^/sLe^x^ oligosaccharide structures, respectively^26,27^.

### Determination of fucose expression in the seminal plasma and blood serum clusterin

Three biotinylated fucose-specific lectins: *Lotus tetragonolobus* agglutinin (LTA, catalog No. B-1325, Vector Laboratories Inc., Burlingame, CA, USA), *Ulex europaeus* agglutinin (UEA, catalog No. B-1065, Vector Laboratories Inc., Burlingame, CA, USA) and *Lens culinaris* agglutinin (LCA, catalog No. B-1045, Vector Laboratories Inc., Burlingame, CA, USA) were used to determine fucose expression in the lectin-ELISA procedure according to the Kratz et al.^24^ with modifications described below. The specificity of lectins is not absolute, and they can react with more than one oligosaccharide residue. *Lotus tetragonolobus* agglutinin and *Ulex europaeus* agglutinin detect fucoses linked to the galactose or antennary N-acetylglucosamine by α1,3 glycosidic bond and α1,2 glycosidic bond, respectively^28^. *Lens culinaris* agglutinin was used for core fucose detection^29^.

### Lectin-ELISA procedure

Schematic representation of the whole lectin-ELISA procedure was shown in Supplementary Fig. 1S.

### ELISA-plate coating

ELISA plates (Nunc MaxiSorp, Thermo Fisher Scientific, Denmark) were coated with goat anti-human clusterin polyclonal antibodies (Invitrogen, Thermo Fisher Scientific, catalog No. PA1-26903; Rockford, USA). The antibodies were diluted 1:5000 in 10 mM TBS, pH = 8.5. After 2 h of incubation at 37 °C, the plate was washed three times with the same buffer. For LCA, due to the high absorbance of blank in the preliminary experiments, oxidation of oligosaccharides of anti-human clusterin polyclonal antibodies, which coated ELISA plate, was performed. Sodium meta-periodate solution (100 mM NaIO_4_, 100 mM NaHCO_3_, pH = 8.1) was added, and after 90-min incubation at room temperature in the dark, the plate was washed with 10 mM TBS pH = 7.5. The next step, free binding sites were blocked by 10 mM TBS, 0.1% Tween20, 1% BSA (blocking buffer, pH = 7.5). After 2 h of incubation at 37 °C, plates were stored at 4 °C overnight. In the lectin-ELISA procedure for LTA and UEA, the step of oligosaccharide oxidation was unnecessary and therefore omitted. After Ab-coating and washing steps, the free binding sites of ELISA wells were blocked by blocking buffer as described above for LCA.

### Sample dilution

Seminal plasma and sera samples were diluted in 10 mM TBS, 0.1% Tween20, pH = 7.5 buffer to obtain proper clusterin concentration per well: 1 ng/100 µL for seminal plasma and 50 ng/100 µL for serum samples. Samples were incubated at 37 °C for two hours with gentle shaking. All samples were analyzed in duplicate to minimize imprecision. Two pairs of blank were added to each lectin-ELISA experiment—they contained all reagents, but instead of patients’ samples, 10 mM TBS, 0.1% Tween20, pH = 7.5 (washing buffer) was used. After each next step of examination, wells were washed using a washing buffer.

### Clusterin reduction

Due to the limited availability of some clusterin glycans, we decided to include one step of the lectin-ELISA procedure and unfold seminal plasma and serum clusterin bound with antibodies, using dithiothreitol (DTT). DTT was diluted in 0.1 M Tris–HCl, pH = 8.0 to obtain concentration 2 mg/mL. After 70 min. incubation at 37 °C plates were washed three times using 10 mM TBS, pH = 7.5. This step was applied in the lectin-ELISA procedure for all lectins used.

### Lectin-fucose interactions

Biotinylated lectins were used to detect α1,3-linked, α1,2-linked antennary fucose, and α1,6-linked core fucose (LTA, UEA, LCA, respectively). All lectins dilutions were established in the series of preliminary experiments using 10 mM TBS containing 1 mM CaCl_2,_ 1 mM MgCl_2_ × 6H_2_O, 1 mM MnCl_2_ × 4H_2_O, 1% BSA and 0.1% Tween20, pH = 7.5. *Lotus tetragonolobus* lectin was diluted 1:100, *Ulex europaeus* agglutinin 1:250, and *Lens culinaris* agglutinin 1:2,000, and next, plates were incubated one hour at 37 °C with gentle shaking.

### Clusterin-lectin complexes detection

ExtrAvidin alkaline phosphatase labeled (Sigma-Aldrich, catalog No. E2636; Saint Louis, USA) diluted 1:10,000 in the washing buffer was used to quantify clusterin-lectin complexes. After 1 h of incubation at 37 °C, the color reaction with disodium para-nitrophenyl phosphate was induced. The absorbances were measured with Mindray MR-96A Microplate Reader (Shenzhen Mindray Bio-Medical Electronics, China) at 405 nm with a reference filter of 630 nm. The results were expressed in the absorbance units (AU) after subtracting the absorbances of the blank samples.

### Statistical analysis

Statistical analysis was performed using Statistica 13.3PL software (StatSoft Inc., Tulsa, OK, USA). Shapiro–Wilk's test was used to analyze normality of all parameters distribution, and values obtained for all relative reactivities with lectins were presented as mean ± SD (SD—standard deviation) and the graphs as median with interquartile range (Q1–Q3). The U Mann–Whitney test was used to compare all relative reactivities with lectins used, and CLU and FUTs concentrations between normozoospermic group versus each group of patients with abnormal semen parameters, and between AT, OAT and T groups. The Spearman’s rank correlation was used to check the associations between measured relative reactivities with lectins, CLU concentrations, and FUTs levels, also for the comparison of both biological materials examined. Moreover, the correlations between selected sperm parameters, being a part of standard ejaculate analysis, and seminal plasma parameters analyzed by us in the study were investigated. The diagnostic significance of lectins relative reactivities with CLU glycans, CLU, and FUTs concentrations was analyzed using receiver operating characteristic (ROC) curves. Based on the AUC, the clinical value of laboratory test can be defined as: 0–0.5—zero, 0.5–0.7—limited, 0.7–0.9—moderate and > 0.9—high^30^. The Youden index method was used for the determination of cut-off points. Moreover, for seminal plasma and serum samples in which all parameters were determined, and only for those parameters for which the AUC values, determined in ROC analysis, were moderate or high, cluster analysis was performed: the results were presented as a dendrogram, beginning from one cluster in which all examined subjects were gathered. The study was performed for 93 seminal plasmas and 83 serum samples for which all selected parameters were determined. All subjects were divided into groups exclusively based on differences or identities in the values of selected factors. In the next step, the subjects were clustered. Patients who presented similarities in terms of the values of all the analyzed traits were grouped together, and a separate cluster was formed for those with different values. To summarize, the greater distance of separation means the greater differences in subject characteristics. The similarities between samples were calculated using an Euclidean metric on the original data points, with no reference to the clinical status of the subjects. The *p*-Values less than 0.05 were considered significant.

## Results

**Figure 1:**
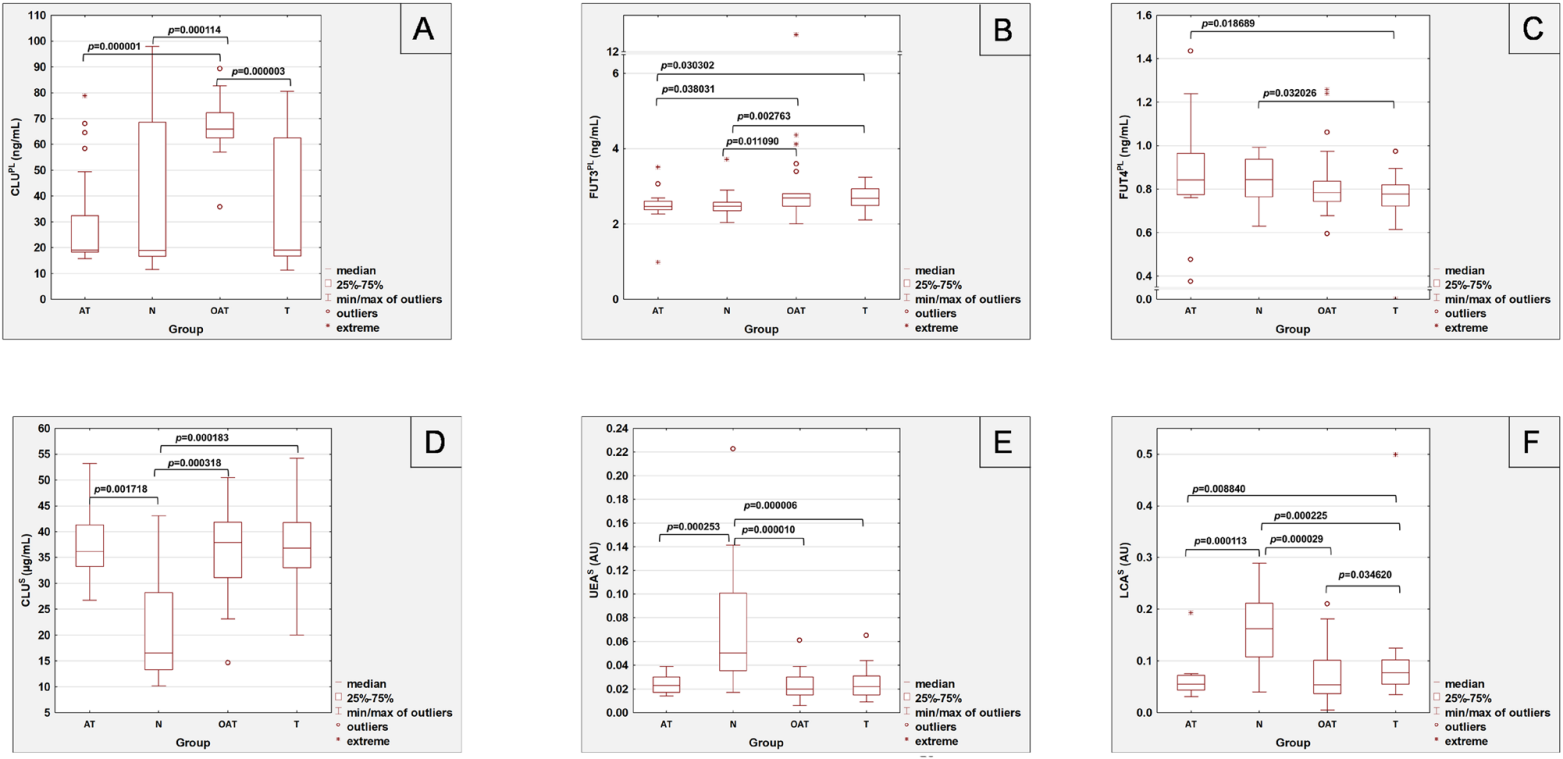
The values of seminal plasma (**A–C**) and serum (**D–F**) parameters measurements. CLU^PL^—seminal plasma CLU concentration; FUT3^PL^—seminal plasma FUT3 concentration; FUT4^PL^—seminal plasma FUT4 concentration; CLU^S^—serum CLU concentration; UEA^S^—relative reactivity of serum CLU glycans with *Ulex europaeus* agglutinin; LCA^S^- relative reactivity of serum CLU glycans with *Lens culinaris* agglutinin. A two-tailed *p*-Value of less than 0.05 was considered significant.

The mean values of analyzed parameters are summarized in Table 1, and Fig. 1 shows the results of determinations only for those parameters whose values significantly differed between the study groups.

**Table 1:** The values of selected parameters measurements in seminal plasma and serum groups of patients with fertility problems.

Parameter	Group			
	N N^PL^ = 29 N^S^ = 16	T N^PL^ = 38 N^S^ = 31	AT N^PL^ = 22 N^S^ = 16	OAT N^PL^ = 29 N^S^ = 27
	MEAN ± SD	MEAN ± SD	MEAN ± SD	MEAN ± SD
CLU^PL^ (ng/mL)	36.46 ± 27.66 *p* = 0.000114**	33.08 ± 23.44 *p* = 0.000003**	29.43 ± 19.95 *p* = 0.000001**	66.59 ± 9.66
CLU^S^ (µg/mL)	21.53 ± 11.91 *p* = 0.001718* *p* = 0.000318** *p* = 0.000183***	37.53 ± 7.20	38.25 ± 7.63	36.73 ± 7.55
FUT3^PL^ (ng/mL)	2.506 ± 0.317 *p* = 0.011090** *p* = 0.002763***	2.698 ± 0.292	2.484 ± 0.506 *p* = 0.038031** *p* = 0.030302***	3.135 ± 1.853
FUT3^S^ (ng/mL)	3.260 ± 1.478	4.422 ± 3.937	4.640 ± 2.724	5.138 ± 4.385
FUT4^PL^ (ng/mL)	0.839 ± 0.110 *p* = 0.032026***	0.757 ± 0.159	0.871 ± 0.250 *p* = 0.018689***	0.822 ± 0.149
FUT4^S^ (ng/mL)	1.120 ± 0.593	1.508 ± 1.423	2.034 ± 1.537	2.246 ± 2.052
LTA^PL^ (AU)	0.647 ± 0.630	0.642 ± 0.497	0.590 ± 0.476	0.648 ± 0.516
LTA^S^ (AU)	0.200 ± 0.124	0.175 ± 0.068	0.170 ± 0.065	0.203 ± 0.118
UEA^PL^ (AU)	0.315 ± 0.230	0.360 ± 0.353	0.485 ± 0.433	0.439 ± 0.414
UEA^S^ (AU)	0.070 ± 0.055 *p* = 0.000253* *p* = 0.00001** *p* = 0.000006***	0.024 ± 0.012	0.024 ± 0.008	0.024 ± 0.011
LCA^PL^ (AU)	0.323 ± 0.220	0.409 ± 0.238	0.416 ± 0.210	0.377 ± 0.239
LCA^S^ (AU)	0.162 ± 0.078 *p* = 0.000113* *p* = 0.000029** *p* = 0.000225***	0.092 ± 0.080	0.064 ± 0.038 *p* = 0.008840***	0.069 ± 0.048 *p* = 0.034620***

CLU^PL^—seminal plasma CLU concentration; CLU^S^—serum CLU concentration; FUT3^PL^—seminal plasma FUT3 concentration; FUT3^S^—serum FUT3 concentration; FUT4^PL^—seminal plasma FUT4 concentration; FUT4^S^—serum FUT4 concentration; LTA^PL^—relative reactivity of seminal plasma CLU glycans with *Lotus tetragonolobus* agglutinin; LTA^S—^relative reactivity of serum CLU glycans with *Lotus tetragonolobus* agglutinin; UEA^PL^—relative reactivity of seminal plasma CLU glycans with *Ulex europaeus* agglutinin; UEA^S^—relative reactivity of serum CLU glycans with *Ulex europaeus* agglutinin; LCA^PL^—relative reactivity of seminal plasma CLU glycans with *Lens culinaris* agglutinin; LCA^S^—relative reactivity of serum CLU glycans with *Lens culinaris* agglutinin. Significant differences versus: *AT group, **OAT group, ***T group. A two-tailed *p*-Value of less than 0.05 was considered significant.

### Clusterin concentration

Seminal plasma clusterin concentrations were significantly higher in the OAT group (median value: 66.01 ng/mL) in comparison to the normozoospermic (median value: 18.98 ng/mL), asthenoteratozoospermic (median value: 19.02 ng/mL) and teratozoospermic group (median value: 19.13 ng/mL) with significances of *p* = 0.000114, *p* = 0.000001 and *p* = 0.000003, respectively. In contrast, CLU concentrations in sera were significantly lower in the normozoospermic group (median value: 16.54 µg/mL) in comparison to the asthenoteratozoospermic (median value: 36.16 µg/mL; *p* = 0.001718), oligoasthenoteratozoospermic (median value: 37.90 µg/mL; *p* = 0.000318) and teratozoospermic (median value: 36.82 µg/mL; *p* = 0.000183) groups (Fig. 1).

### Fucosyltransferases concentrations

The concentrations of seminal plasma FUT3 which participate in the formation of variety of Le oligosaccharide structures in glycoproteins, were significantly higher in teratozoospermic patients (median value: 2.685 ng/mL) in comparison to the normozoospermic (median value: 2.470 ng/mL) and asthenoteratozoospermic (median value: 2.462 ng/mL) samples with significances of *p* = 0.002763 and *p* = 0.030302, respectively. Seminal plasma FUT3 concentrations were also significantly higher in the OAT patients (median value: 2.690 ng/mL) in comparison to the AT group (median value: 2.462 ng/mL; *p* = 0.038031) and N group (median value: 2.470 ng/mL; *p* = 0.011090) (Table 1).

The concentrations of seminal FUT4, which may be associated with the expression of i.a. Le^x^/sLe^x^ oligosaccharide glycan structures, were significantly higher in the normozoospermic samples (median value: 0.843 ng/mL) in comparison to the teratozoospermic group (median value: 0.777 ng/mL) with a significance of *p* = 0.032026. Moreover, FUT4 concentrations in AT group (median value: 0.843 ng/mL) were significantly higher than in the T group with a significance of *p* = 0.018689. No significant differences for serum FUT3 and FUT4 concentrations between groups analyzed by us were found (Table 1). For serum FUT3 concentrations, the median values were following: 2.923; 2.821; 3.499 and 2.838 ng/mL in normozoospermia, T, AT and OAT groups, respectively. The median values of FUT4 concentrations were: 1.080; 1.013; 1.462 and 1.013 ng/mL in normozoospermia, T, AT and OAT groups, respectively.

### Fucose expression in the glycans of serum and seminal plasma clusterin

There were no significant differences between seminal plasma groups in relative reactivities of CLU glycans with fucose-specific lectins LTA, UEA, and LCA (Table 1). The median value of LTA relative reactivity with seminal CLU glycans was 0.371 AU for normozoospermia, and 0.542; 0.439 and 0.653 AU, for T, AT and OAT groups, respectively. The median values of UEA relative reactivity with CLU glycans were the following: 0.283 AU in normozoospermia, 0.284; 0.283 and 0.277 AU in T, AT and OAT groups, respectively. The median value of LCA relative reactivity with seminal CLU glycans was 0.276 AU for normozoospermic group, and 0.341; 0.415 and 0.326 AU for T, AT and OAT groups, respectively.

No significant differences were observed between the studied groups in the values of relative reactivities of serum CLU glycans with LTA, and the medians of the obtained values were as follows: 0.171 AU in normozoospermia, 0.144 AU in teratozoospermia, 0.160 AU for asthenoteratozoospermia and 0.174 AU in oligoasthenoteratozoospermia. Relative reactivities of CLU glycans with UEA in sera of normozoospermic patients (median value: 0.05 AU) were significantly higher in comparison to the other groups: AT (median value: 0.023 AU; *p* = 0.000253), OAT (median value: 0.020 AU; *p* = 0.00001) and T (median value: 0.022 AU; *p* = 0.000006). Similar dependency was observed for relative reactivity of CLU glycans with LCA in serum samples—normozoospermic patients (median value: 0.162 AU) had significantly higher core-fucose expression in comparison to the AT (median value: 0.055 AU; *p* = 0.000113), OAT (median value: 0.054 AU; *p* = 0.000029) and T (median value: 0.077 AU; *p* = 0.000225) groups. Additionally, the relative reactivity of serum CLU glycans with LCA was significantly higher in the T group compared to the OAT and AT groups, with the significance of *p* = 0.03462 and *p* = 0.00884, respectively (Table 1). The results of correlations between values of analyzed parameters are summarized in Table 2.

**Table 2:** The correlations between values of analyzed parameters.

Parameter	CLU^PL^ (ng/mL)	CLU^S^ (µg/mL)	FUT3^PL^ (ng/mL)	FUT3^S^ (ng/mL)	FUT4^PL^ (ng/mL)	FUT4^S^ (ng/mL)	LTA^PL^ (AU)	LTA^S^ (AU)	UEA^PL^ (AU)	UEA^S^ (AU)	LCA^PL^ (AU)
CLU^S^ (µg/mL)	NS										
FUT3^PL^ (ng/mL)	*r* = 0.251 *p* = 0.039										
FUT3^S^ (ng/mL)		NS	*r* = 0.352 *p* = 0.003								
FUT4^PL^ (ng/mL)	NS		NS								
FUT4^S^ (ng/mL)		NS		*r* = 0.949 *p* < 0.001	*r* = 0.361 *p* = 0.03						
LTA^PL^ (AU)	NS		NS		NS						
LTA^S^ (AU)		*r* = − 0.338 *p* = 0.005		*r* = 0.276 *p* = 0.023		*r* = 0.300 *p* = 0.013	NS				
UEA^PL^ (AU)	NS		*r* = − 0.341 *p* = 0.004		NS		*r* = 0.768 *p* < 0.001				
UEA^S^ (AU)		NS		NS		NS		NS	*r* = 0.288 *p* = 0.017		
LCA^PL^ (AU)	NS						*r* = 0.833 *p* < 0.001		*r* = 0.651 *p* < 0.001		
LCA^S^ (AU)		NS						NS		*r* = 0.392 *p* = 0.001	NS

CLU^PL^—seminal plasma CLU concentration; CLU^S^—serum CLU concentration; FUT3^PL^—seminal plasma FUT3 concentration; FUT3^S^—serum FUT3 concentration; FUT4^PL^—seminal plasma FUT4 concentration; FUT4^S^—serum FUT4 concentration; LTA^PL^—relative reactivity of seminal plasma CLU glycans with *Lotus tetragonolobus* agglutinin; LTA^S^—relative reactivity of serum CLU glycans with *Lotus tetragonolobus* agglutinin; UEA^PL^—relative reactivity of seminal plasma CLU glycans with *Ulex europaeus* agglutinin; UEA^S^—relative reactivity of serum CLU glycans with *Ulex europaeus* agglutinin; LCA^PL^—relative reactivity of seminal plasma CLU glycans with *Lens culinaris* agglutinin; LCA^S^—relative reactivity of serum CLU glycans with *Lens culinaris* agglutinin. NS—not significant. A two-tailed *p*-Value of less than 0.05 was considered significant.

### Comparison of the fucosylation profile between seminal plasma and serum

**Figure 2:**
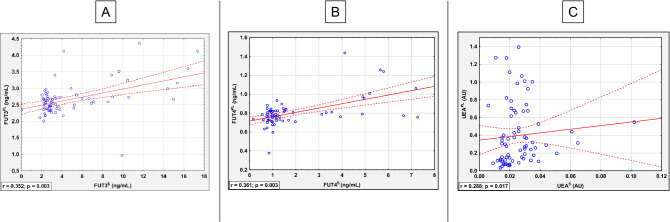
Comparison of glycosylation profile in two biological fluids (seminal plasma and serum) in the study group (**A–C**). FUT3^PL^—seminal plasma FUT3 concentration; FUT3^S^—serum FUT3 concentration; FUT4^PL^—seminal plasma FUT4 concentration; FUT4^S^—serum FUT4 concentration; UEA^PL^—relative reactivity of CLU glycans with *Ulex europaeus* agglutinin in seminal plasma; UEA^S^—relative reactivity of CLU glycans with *Ulex europaeus* agglutinin in serum. The correlations were calculated using the Spearman rank test and a two-tailed *p*-Value of less than 0.05 was considered significant. The dashed line points 95% of confidence interval.

The comparison of profile and degree of CLU glycans fucosylation in two biological fluids obtained from the same patient, blood serum and seminal plasma, enabled to demonstrate a weak positive correlation in relative reactivity of CLU glycans with UEA (*r* = 0.288, *p* = 0.018), however, no other associations in fucosylation of seminal plasma and serum clusterin glycans were found. We also found weak positive correlations in concentrations of fucosyltransferases between both examined biological fluids (FUT4: *r* = 0.361, *p* = 0.003; FUT3: *r* = 0.352, *p* = 0.003) (Fig. 2, Table 2).

### Correlation analysis between selected semen parameters and seminal plasma parameters

The correlations between selected semen parameters, being a part of standard ejaculate analysis, and seminal plasma parameters analyzed by us are shown in the Supplementary Table 1S. Seminal plasma CLU concentration demonstrated moderate negative correlation with total sperm count (*r* = − 0.409; *p* = 0.001) and a weak negative correlation with the percentage of sperm with progressive motility (*r* = − 0.317; *p* = 0.009). Seminal plasma FUT3 concentration weakly negatively correlated with the percentage of sperm with normal morphology (*r* = − 0.300*; p* = 0.014). A weak positive correlation between relative reactivity of seminal plasma CLU glycans with *Ulex europaeus* agglutinin and the percentage of sperm with normal morphology was also found (*r* = 0.270; *p* = 0.027) (Supplementary Table 1S).

### ROC curve analysis

**Figure 3:**
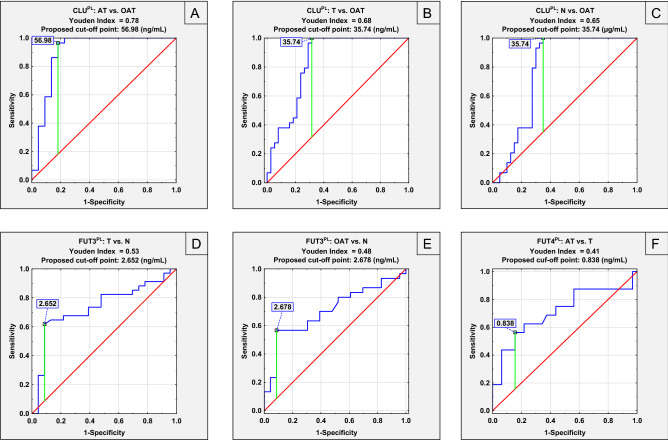
Receiver operating characteristic (ROC) curves for seminal plasma parameters with the area under the curve (AUC) higher than 0.700 (**A–F**). CLU^PL^—seminal plasma CLU concentration; FUT3^PL^—seminal plasma FUT3 concentration; FUT4^PL^—seminal plasma FUT4 concentration.

**Figure 4:**
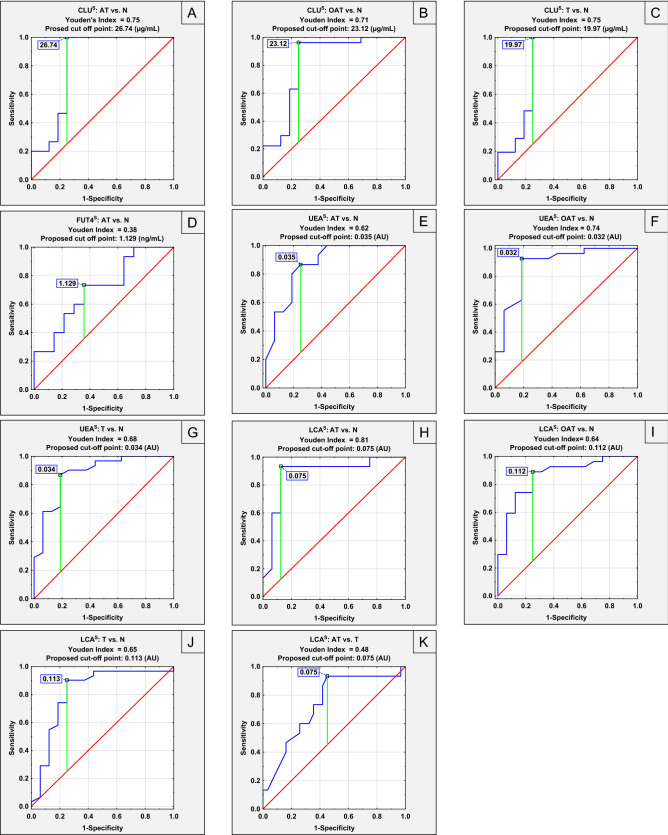
Receiver operating characteristic (ROC) curves for serum parameters with the area under the curve (AUC) more than 0.700 (**A–K**). CLU^S^—serum CLU concentration; FUT4^S^—serum FUT4 concentration; UEA^S^-relative reactivity of serum CLU glycans with *Ulex europaeus* agglutinin; LCA^S^-relative reactivity of serum CLU glycans with *Lens culinaris* agglutinin.

We performed receiver operating characteristic curves for all seminal plasma and serum parameters determined by us (Tables 3, 4, respectively). However, in Figs. 3 and 4, the results of ROC curve analysis were shown for seminal plasma and serum parameters, respectively, for which the area under the curve (AUC) was higher than 0.7. The cut-off points determined using the Youden index, are presented in Table 3 (seminal plasma) and Table 4 (serum).

**Table 3:** Summary of receiver operating characteristic (ROC) curves for seminal plasma parameters.

Parameter	Group		AUC	AUC with 95% confidence interval	Cut off point	Sensitivity	Specificity	*P*
CLU^PL^	AT	vs. N	0.494	0.352–0.637	68.01	0.955	0.275	0.938
	**OAT**		**0.774**	**0.658–0.890**	**35.74**	**1.000**	**0.650**	**0.000**
	T		0.495	0.366–0.625	17.71	0.711	0.400	0.945
	AT vs. T		0.502	0.356–0.649	58.41	0.864	0.290	0.975
	**OAT vs. T**		**0.834**	**0.736–0.932**	**35.74**	**1.000**	**0.684**	**0.000**
	**OAT vs. AT**		**0.903**	**0.801–1.000**	**56.98**	**0.966**	**0.818**	**0.000**
FUT3^PL^	AT	vs. N	0.541	0.352–0.729	2.38	0.875	0.304	0.672
	**OAT**		**0.706**	**0.564–0.847**	**2.68**	**0.568**	**0.913**	**0.004**
	**T**		**0.736**	**0.601–0.871**	**2.65**	**0.618**	**0.913**	**0.001**
	AT vs. T		0.691	0.527–0.855	2.62	0.813	0.618	0.022
	OAT vs. T		0.509	0.363–0.656	3.40	0.233	1.000	0.901
	OAT vs. AT		0.686	0.529–0.844	2.68	0.567	0.813	0.021
FUT4^PL^	AT	vs. N	0.536	0.335–0.738	1.01	0.188	1.000	0.722
	OAT		0.614	0.436–0.792	0.82	0.714	0.667	0.210
	T		0.685	0.515–0.855	0.82	0.813	0.667	0.033
	**AT vs. T**		**0.710**	**0.539–0.881**	**0.84**	**0.563**	**0.844**	**0.016**
	OAT vs. T		0.574	0.428–0.720	0.81	0.464	0.710	0.323
	OAT vs. AT		0.647	0.468–0.826	0.82	0.714	0.625	0.107
LTA^PL^	AT	vs. N	0.448	0.268–0.628	1.02	0.875	0.333	0.572
	OAT		0.509	0.347–0.671	0.65	0.517	0.667	0.917
	T		0.559	0.398–0.720	0.12	0.971	0.250	0.474
	AT vs. T		0.517	0.349–0.684	0.34	0.500	0.647	0.847
	OAT vs. T		0.490	0.342–0.638	0.69	0.483	0.677	0.893
	OAT vs. AT		0.500	0.329–0.671	0.72	0.448	0.813	1.000
UEA^PL^	AT	vs. N	0.583	0.394–0.773	0.79	0.313	0.958	0.389
	OAT		0.510	0.350–0.670	0.67	0.345	0.917	0.902
	T		0.492	0.340–0.644	0.62	0.206	0.917	0.918
	AT vs. T		0.573	0.394–0.751	0.79	0.313	0.941	0.426
	OAT vs. T		0.508	0.355–0.660	0.74	0.276	0.941	0.922
	OAT vs. AT		0.574	0.404–0.745	0.11	0.345	0.875	0.392
LCA^PL^	AT	vs. N	0.651	0.474–0.827	0.33	0.688	0.682	0.095
	OA		0.567	0.407–0.726	0.32	0.552	0.682	0.414
	T		0.618	0.466–0.770	0.33	0.594	0.682	0.129
	AT vs. T		0.467	0.300–0.633	0.53	0.875	0.344	0.696
	OAT vs. T		0.462	0.315–0.608	0.43	0.448	0.625	0.609
	OAT vs. AT		0.563	0.395–0.730	0.16	0.276	1.000	0.465

CLU^PL^—seminal plasma CLU concentration; FUT3^PL^—seminal plasma FUT3 concentration; FUT4^PL^—seminal plasma FUT4 concentration; LTA^PL^—relative reactivity of seminal plasma CLU glycans with *Lotus tetragonolobus* agglutinin; UEA^PL^—relative reactivity of seminal plasma CLU glycans with *Ulex europaeus* agglutinin; LCA^PL^—relative reactivity of seminal plasma CLU glycans with *Lens culinaris* agglutinin. Area under the ROC curve (AUC) is given with 95% confidence interval. Data with AUC equal or greater than 0.706 are marked in bold. Based on the AUC, the clinical value of laboratory test can be defined as: 0–0.5—zero, 0.5–0.7—limited, 0.7–0.9—moderate and > 0.9—high.

**Table 4:** Summary of receiver operating characteristic (ROC) curves for serum parameters.

Parameter	Group		AUC	AUC with 95% confidence interval	Cut off point	Sensitivity	Specificity	*P*
CLU^S^	**AT**	**vs. N**	**0.821**	**0.657–0.985**	**26.74**	**1.000**	**0.750**	**0.000**
	**OAT**		**0.819**	**0.668–0.971**	**23.12**	**0.963**	**0.750**	**0.000**
	**T**		**0.823**	**0.665–0.981**	**19.97**	**1.000**	**0.750**	**0.000**
	AT vs. T		0.503	0.321–0.685	45.66	0.200	0.903	0.972
	OAT vs. T		0.497	0.345–0.649	37.29	0.623	0.516	0.969
	OAT vs. AT		0.494	0.305–0.683	36.77	0.667	0.533	0.949
FUT3^S^	AT	vs. N	0.667	0.463–0.870	2.41	1.000	0.308	0.109
	OAT		0.541	0.346–0.737	4.17	0.333	0.923	0.679
	T		0.521	0.333–0.710	4.90	0.290	0.923	0.826
	AT vs. T		0.615	0.452–0.778	2.41	1.000	0.290	0.167
	OAT vs. T		0.555	0.405–0.704	6.65	0.296	0.871	0.471
	OAT vs. AT		0.574	0.399–0.749	3.32	0.667	0.600	0.407
FUT4^S^	**AT**	vs. N	**0.710**	**0.520–0.899**	**1.13**	**0.733**	**0.643**	**0.030**
	OAT		0.619	0.431–0.808	0.77	1.000	0.357	0.216
	T		0.554	0.364–0.745	0.80	0.862	0.357	0.577
	AT vs. T		0.634	0.456–0.813	1.19	0.667	0.655	0.134
	OAT vs. T		0.591	0.440–0.741	0.92	0.889	0.345	0.238
	OAT vs. AT		0.535	0.348–0.721	1.09	0.556	0.733	0.717
LTA^S^	AT	vs. N	0.569	0.362–0.776	0.18	0.733	0.500	0.515
	OAT		0.543	0.364–0.721	0.14	0.741	0.438	0.638
	T		0.537	0.362–0.712	0.16	0.613	0.563	0.676
	AT vs. T		0.485	0.304–0.666	0.16	0.533	0.581	0.871
	OAT vs. T		0.580	0.430–0.730	0.14	0.741	0.484	0.295
	OAT vs. AT		0.584	0.402–0.765	0.14	0.815	0.400	0.365
UEA^S^	**AT**	**vs. N**	**0.869**	**0.742–0.995**	**0.04**	**0.867**	**0.750**	**0.000**
	**OAT**		**0.878**	**0.764–0.993**	**0.03**	**0.926**	**0.813**	**0.000**
	**T**		**0.879**	**0.771–0.987**	**0.03**	**0.871**	**0.813**	**0.000**
	AT vs. T		0.539	0.366–0.711	0.02	0.933	0.258	0.661
	OAT vs. T		0.520	0.369–0.670	0.03	0.926	0.226	0.797
	OAT vs. AT		0.542	0.359–0.725	0.02	0.259	0.933	0.653
LCA^S^	**AT**	**vs. N**	**0.881**	**0.746–1.000**	**0.08**	**0.933**	**0.875**	**0.000**
	**OAT**		**0.861**	**0.745–0.977**	**0.11**	**0.889**	**0.750**	**0.000**
	**T**		**0.818**	**0.670–0.965**	**0.11**	**0.903**	**0.750**	**0.000**
	**AT vs T**		**0.737**	**0.582–0.891**	**0.08**	**0.933**	**0.548**	**0.003**
	OAT vs. T		0.662	0.515–0.810	0.05	0.519	0.839	0.030
	OAT vs. AT		0.493	0.317–0.668	0.08	0.333	0.933	0.934

CLU^S^—serum CLU concentration; FUT3^S^—serum FUT3 concentration; FUT4^S^—serum FUT4 concentration; LTA^S^—relatsive reactivity of serum CLU glycans with *Lotus tetragonolobus* agglutinin; UEA^S^—relative reactivity of serum CLU glycans with *Ulex europaeus* agglutinin; LCA^S^—relative reactivity of serum CLU glycans with *Lens culinaris* agglutinin. Area under the ROC curve (AUC) is given with 95% confidence interval. Data with AUC equal or greater than 0.710 are marked in bold. Based on the AUC, the clinical value of laboratory test can be defined as: 0–0.5—zero, 0.5–0.7—limited, 0.7–0.9—moderate and > 0.9—high.

### Cluster analysis

For the cluster analysis were selected parameters that in the ROC curve analysis had moderate or high clinical value (AUC ≥ 0.706 for seminal plasma samples and AUC ≥ 0.710 for serum samples). Three parameters were selected for seminal plasma to perform the cluster analysis: CLU, FUT3, FUT4 concentrations, and four parameters for serum: CLU and FUT4 concentrations, relative reactivities with UEA and LCA. The study was performed for 93 seminal plasmas and 83 serum samples for which all selected parameters were determined. All subjects were divided into groups exclusively based on differences or identities in the values of selected factors.

In seminal plasma, at 90% distance, all samples could be regarded as homogenous formation. The first cluster was distinguished at 42% distance as a group composed of 42 samples. The second cluster could be distinguished at 21% distance as a group of 44 seminal plasma samples, 25 of which were from the OAT group (89% of all OAT seminal plasma samples). The third cluster could be distinguished at 16% distance and comprised 7 subjects (Supplementary Fig. 2S).

For serum, at 88% distance, all samples were regarded as homogenous formation. The first cluster could be distinguished at 49% distance as a group of 54 subjects. The second cluster could be separated at 26% distance as 11 samples, containing 8 of 12N samples (67% of all N subjects). The third cluster was distinguished at 16% distance, containing 18 serum samples from AT, OAT and T groups (Supplementary Fig. 3S).

## Discussion

### Clusterin concentration

Based on the results concerning seminal plasma CLU concentration, we can conclude that the observed significant differences between N, T, and AT groups versus the OAT group may be associated with the increase of CLU production in the male reproductive organs and corresponds with the lowered total number of spermatozoa in the ejaculate. The above finding was additionally confirmed by observed moderate negative correlation between seminal CLU concentration and total sperm count in the semen. Our findings stand in contradiction to the research of Fukuda et al., in which the normozoospermic seminal plasma samples of infertile men had significantly higher CLU concentration (47.9 ± 20.9 ng/mL) in comparison to the oligozoospermic group of patients (28.2 ± 8.6 ng/mL)^31^. On the other hand, Salehi et al. reported higher seminal plasma CLU concentrations in the fertile men (48.3 ± 38.59 ng/mL) in comparison to the infertile group (14.48 ± 9.74 ng/mL) without particular classification based on the sperm abnormalities^32^. Interestingly, serum CLU concentration values were significantly decreased in the normozoospermic group compared to T, AT, and OAT groups. The observed by us different expression of CLU present in two examined biological fluids is apparently related to the distinct location of its synthesis and the biological role it plays in various parts of male organism.

### Fucosyltransferases concentration

A comparison of the seminal plasma FUT3 concentration between T and AT groups suggests that asthenozoospermia may be associated with decreasing FUT3 concentration in this biological fluid. Some authors have demonstrated that seminal plasma FUTs are very important during interactions between sperm surface and zona pellucida. Chiu et al.^33^ showed that seminal plasma FUT5 inhibits this process through binding with glycodelin A. The co-immunoprecipitation of FUT3, FUT5, and glycodelin A confirmed the presence of these enzymes in the sperm cells’ membrane^33^. Human seminal plasma fucosyltransferases are interesting and need to be investigated, especially in the context of their influence on the expression of Lewis oligosaccharide structures. Decreased total number of spermatozoa in the ejaculate, together with increased expression of sperm malformations, seem to be associated with the elevated expression of wide spectrum of Le oligosaccharide structures in seminal plasma glycoproteins synthesized within the male reproductive tract, which was demonstrated by a significant increase of FUT3 concentration in OAT and T groups versus N, with no differences between N and AT groups; however, this hypothesis should be confirmed in the future study, focused also on analysis of FUTs expression, which specificity of action is not so wide. There is no current literature data concerning FUT3 concentrations in human seminal plasma. Our previous study on seminal plasma AGP (α1-acid glycoprotein) glycosylation showed, that Le^a^ expression on AGP coincides with a higher degree of glycans branching and a relative increased α4-fucosyltransferase activity^34^. In other study, we reported that the expression of Le^y^ structures was significantly increased in selected glycoprotein bands detected in lectin-blotting of normozoospermic seminal plasmas compared to the control group of men with proved fertility. However, it should be underlined that in the present study, we examined patients with a variety of sperm abnormalities, and distinct methods were used for the analysis of Le^y^ oligosaccharide structure expression^35^. In the present study, we also did not have the opportunity to compare the results obtained for infertile patients with those obtained for the normozoospermic group of fertile men. Pang et al.^36^ investigated the expression of Le^x^ and Le^y^ oligosaccharide structures in sperm cells’ N-glycans of normozoospermic infertile men and healthy sperm bank donors. The authors concluded that defective sperm had a high level of Le^y^ expression, and additionally the localization of these structures was distinct than in the case of the control group^36^, what is in accordance with our findings for seminal plasma FUTs levels, which significantly elevated for FUT3 and decreased for FUT4 in T group versus N subjects, if we take into account that FUT3 influence the formation of, among others Le^x^ and Le^y^ oligosaccharide structures, and FUT4 the formation of inter alia Le^x^ structures.

The comparison the seminal plasma FUT4 concentrations between study groups indicates that spermatozoa morphology abnormalities characteristic for teratozoospermia are associated with decreased FUT4 concentration. Moreover, the observed significant differences in seminal plasma FUT4 concentrations between the T and AT group suggest that motility abnormalities of sperm cells together with morphology disorders may be associated with the relative increase in the expression of Le^a/x^ structures in glycans of seminal plasma glycoproteins. It is worth noting that the differences in seminal plasma FUT4 concentration values between the normozoospermic group compared to the OAT, AT, and T groups, are the opposite of the concentration values of FUT3. In contrast, the expression of Le^x^ structures examined by Kałuża et al. in the asthenozoospermic patients was moderately decreased, compared to the control group for two from five glycoprotein bands. However, the control group investigated by authors was composed of normozoospermic fertile men^35^. Authors found no differences in the expression of Le^x^-decorated glycans in the seminal plasma glycoproteins between the asthenozoospermic group and normozoospermic patients with fertility problems^35^. Still, it should be mentioned that lectin-blotting analysis of glycoprotein bands, separated previously in SDS-PAGE, without detailed analysis of specific glycoprotein, significantly differs in its idea from lectin-ELISA in which specific glycoprotein may be analyzed, and thus the comparison of results obtained is difficult and may be problematic. The presence of Le^x^ and Le^y^ oligosaccharide structures as the DC-SIGN ligands in human seminal plasma glycome was investigated by Clark et al.^37,38^. The authors showed that one of the three major endogenous DC-SIGN ligands in seminal plasma is clusterin, expressing Le^x^ and Le^y^ oligosaccharide structures in the antennary part of its glycans^38^. This conclusion seems to be confirmed by the study conducted by Sabatte et al., who reported that seminal plasma, not serum CLU, is a DC-SIGN ligand^39^. Based on our findings and literature data, we can conclude that any alterations in the expression of Le^x^ and Le^y^ oligosaccharide structures, influenced among others by FUT4 and FUT3, may be responsible for disturbances in intermolecular interactions within the male reproductive system.

The relevance of FUT3 and FUT4 concentration values in decreased male fertility was confirmed by correlation analysis. To the best of our knowledge, our study showed for the first time a weak positive correlation between biological fluids examined for FUT3 (*r* = 0.352, *p* = 0.003) and FUT4 (*r* = 0.361, *p* = 0.003) concentrations (Fig. 2). Additionally, serum FUT3 and FUT4 concentrations demonstrated a very strong positive correlation (*r* = 0.949, *p* < 0.001) what may be explained by similarities in mechanisms stimulating of their action. A weak negative correlation between seminal plasma FUT3 concentration and the relative reactivity of seminal plasma CLU glycans with UEA (*r* = − 0.341, *p* = 0.004) may suggests lowered expression of α1,2-linked fucose being a part of Le^y^ oligosaccharide structure which is compensated by the expression of Le^b^ and/or Le^a^ oligosaccharide structures. Saraswat et al.^25^ confirmed that among branch fucosylated seminal plasma glycoproteins also occur Le^a^ and less frequently Le^b^ glyco-motifs, which may be also present in CLU glycans. However, further investigations in this field are needed. In contrast to the above, serum FUT4 concentration weak positively correlated with the relative reactivity of serum CLU glycans with LTA (*r* = 0.300, *p* = 0.013). It is consistent with the specificity of LTA, which detects fucose linked to galactose via α1,3 glycosidic bound, present in the antennary part of N-glycans, forming Le^x^ oligosaccharide structure, and with the biological function of FUT4, which is responsible for i.a. Le^x^ glycan structures formation^26,28^.

### Fucose expression in the glycans of clusterin present in seminal plasma and serum

Lack of differences in relative reactivities of CLU glycans with fucose-specific lectins between infertile normozoospermic seminal plasma group and groups of patients with abnormal semen parameters indicates that CLU fucosylation is independent of sperm count, progression, and morphology. Future studies should check if any differences exist in seminal CLU fucosylation status between normozoospermic infertile patients with idiopathic infertility and a group of normozoospermic subjects with confirmed fertility. The lack of a normozoospermic group of men with proven fertility is a limitation of our study.

On the other hand, the observed relative reactivities of CLU glycans with UEA in sera may led to the conclusion that changes in α1,2-linked fucose expression observed in serum CLU glycans of infertile men are related to the results of routine seminal analyses, because the expression of fucose being a part of Le^y^ oligosaccharide structures in the CLU glycans was significantly higher in the normozoospermic group. It seems that the expression of UEA-reactive fucose in CLU could be used as a marker of male infertility caused by sperm cells disorders. Based on the results concerning relative reactivity of CLU glycans with LCA in sera, we observed that expression of α1,6-linked core fucose in the CLU glycans in the normozoospermic group is significantly higher than in the other groups characterized by semen abnormalities. Moreover, observed significant differences in LCA reactivity between T and OAT, and between T and AT groups may indicate that complex spermatozoa abnormalities, including more than one disorder, are reflected in the decrease of core fucose expression in CLU glycans. It is also worth noting that, in opposite to serum CLU fucosylation, the expression in glycans of seminal plasma CLU of α1,2-linked fucose, being a part of Le^y^ oligosaccharide structures, as well as α1,6-linked core fucose, was noticeably lower (insignificant, however) in normozoospermic subjects than in the rest of investigated groups of patients, what may suggest the differences in mechanisms involved in glycosylation pattern formation of CLU in seminal plasma and blood serum.

Based on the presence of negative correlation between the expression in glycans of serum CLU LTA-reactive α1,3-linked fucose and serum CLU concentration, we came to the presumption that high concentration of serum CLU may be accompanied by decreased expression of Le^x^ oligosaccharide structures; however, it should be taken into account, that the decreased expression of α1,3-linked fucose may be compensated by increased expression of α1,4-linked fucose typical for Le^a^ oligosaccharide structures and/or by fucose of Le^b^ structures. The above hypothesis needs to be checked in future studies.

As far as we know, this is the first study based on lectin-ELISA measurements concerning the fucosylation analysis of seminal plasma and serum clusterin in men with fertility problems. Lectin-based ELISA is a good research tool because the reactions between lectins and sugar residues in vitro reflect those that occur between sugar residues of glycoprotein glycans and their corresponding ligands in a living organism. In our previous studies in the leukocytospermic patients we examined the fucosylation profile and degree of some other seminal plasma glycoproteins, fibronectin (FN) and AGP^24^. Like CLU, these glycoproteins influence male fertility, and FN, similarly to CLU, participates in the acrosome reaction^40^. Kałuża et al.^35^ evaluated the expression of glycoepitopes that may be important in the context of fetoembryonic defense and confirmed that clusterin is one of the crucial importance. They reported that the relative reactivity of seminal plasma CLU with LTA was significantly lower in infertile patients than in the control group^35^. Olejnik et al., based on their research, suggest that abundant fucosylation of some seminal plasma glycoproteins may bother the normal fertilization process^41^. Further analysis concerning the glycosylation profile of CLU seems to be particularly relevant in the context of oxidative stress and chaperone activity of CLU. Merlotti et al. confirmed that fucosylated seminal plasma CLU performs its chaperone activity and directs damaged proteins to the dendritic cells through DC-SIGN^20^. It will be also interesting to examine the expression of Le^a^ and Le^b^ expression on antennary part of CLU glycans and the comparison of obtained results with the present findings.

### Comparison of the glycosylation profile in both biological fluids

Lack of correlations between relative reactivity of seminal plasma and serum CLU glycans with LTA evidently indicate and confirm that seminal plasma and serum CLU is synthesized and glycosylated in different places of the male organism, which results in differences in CLU glycans composition and fucosylation intensity, especially in the Le^x^ structures formation and core fucosylation, in those two biological fluids.

Our study also showed that exists a strong positive correlation (*r* = 0.768, *p* < 0.001) between LTA and UEA relative reactivities of seminal plasma CLU glycans, probably due to the fact that UEA can detect fucose bounded via α1,2-glycosidic linkage, being a part of bifucosylated structures additionally containing LTA-reactive fucose linked by α1,3-glycosidic bound^42^. Comparison of the glycosylation profile of seminal plasma and serum CLU glycans also showed a weak positive correlation between relative reactivity of CLU glycans with UEA (*r* = 0.288, *p* = 0.017) what may suggest similarity in the relative expression of antennary α1,2-linked fucose in glycans of seminal plasma and serum CLU (Fig. 2).

### ROC curve analysis

It should be underlined that, as far as we know, this is the first study in which simultaneously seminal plasma and serum CLU, FUT3, FUT4 concentrations as well as the relative expression of fucose in CLU glycans were analyzed in the context of decreased male fertility. The results of ROC curve analysis showed that the determination of seminal plasma parameters: CLU, FUT3, and FUT4 concentrations may be helpful in the differentiation of infertile groups of patients with both normal and abnormal seminal parameters (Fig. 3, Table 3). In this study, seminal plasma clusterin concentration has a moderate clinical value and enabled differentiation of OAT group from the normozoospermic group with sensitivity and specificity of 100% and 65%, respectively (proposed cut off point: 35.74 ng/mL, AUC = 0.774) and from the teratozoospermic group with sensitivity and specificity of 100% and 68.4%, respectively (proposed cut-off point: 35.74 ng/mL, AUC = 0.834), but CLU concentration has a high clinical value when differentiating OAT patients from asthenoteratozoospermic men with sensitivity and specificity of 96.6% and 81.8%, respectively (proposed cut off point: 56.98 ng/mL, AUC = 0.903). The above differences most probably are the results of decreased sperm count in the OAT group, which may be caused among others by destructive action on spermatogenesis of oxidative stress accompanying high levels of CLU. FUT3 concentration has a moderate clinical value when to distinguish OAT group from the normozoospermic group with sensitivity and specificity of 56.8% and 91.3%, respectively (proposed cut off point: 2.68 ng/mL, AUC = 0.706) and T group from the normozoospermic group with sensitivity and specificity of 61.8% and 91.3%, respectively (proposed cut off point: 2.65 ng/mL, AUC = 0.736), what let us concluding that for observed differences the strongest influence has together decreased sperm count and increased expression of their malformations. FUT4 concentration with moderate clinical value was useful in the differentiation between AT and T groups with sensitivity and specificity of 56.3% and 84.4%, respectively (proposed cut-off point: 0.84 ng/mL, AUC = 0.710), and the decreased motility of sperm cells was crucial here.

ROC curve analysis for examined by us serum parameters revealed that CLU and FUT4 concentrations and relative reactivity of serum CLU glycans with UEA and LCA may be useful in the differentiation of groups of infertile men with abnormal and normal semen parameters (Fig. 4, Table 4). Serum clusterin concentration enabled differentiation with moderate clinical value the normozoospermic group from: AT group with sensitivity and specificity of 100% and 75%, respectively (proposed cut off point: 26.74 µg/mL, AUC = 0.821), OAT group with sensitivity and specificity of 96.3% and 75%, respectively (proposed cut off point: 23.12 µg/mL, AUC = 0.819) and T group with sensitivity and specificity of 100% and 75%, respectively (proposed cut off point: 19.97 µg/mL, AUC = 0.823). FUT4 concentration also has moderate clinical value and can be used to differentiate asthenoteratozoospermic patients from normozoospermic men with sensitivity and specificity of 73.3% and 64.3%, respectively (proposed cut-off point: 1.13 ng/mL, AUC = 0.710). As in the case for serum CLU concentration, relative reactivity of serum CLU glycans with UEA have moderate clinical value and enabled differentiation of normozoospermic patients from: AT patients with sensitivity and specificity of 86.7% and 75%, respectively (proposed cut off point: 0.04 AU, AUC = 0.869), OAT patients with sensitivity and specificity of 92.6% and 81.3%, respectively (proposed cut off point: 0.03 AU, AUC = 0.878) and T patients with sensitivity and specificity of 87.1% and 81.3%, respectively (proposed cut off point: 0.03 AU, AUC = 0.879). Relative reactivity of serum CLU glycans with LCA with moderate clinical value make possible the differentiation of normozoospermic patients from: AT patients with sensitivity and specificity of 93.3% and 87.5%, respectively (proposed cut off point: 0.08 AU, AUC = 0.881), OAT patients with sensitivity and specificity of 88.9% and 75%, respectively (proposed cut off point: 0.11 AU, AUC = 0.861) and T patients with sensitivity and specificity of 90.3% and 75%, respectively (proposed cut off point: 0.11 AU, AUC = 0.818). Moreover, this parameter, which has moderate clinical value, also differentiated teratozoospermic and asthenoteratozoospermic patients with sensitivity and specificity of 93.3% and 54.8%, respectively (proposed cut-off point: 0.08 AU, AUC = 0.737), what is most likely due to reduced sperm cells motility.

### Cluster analysis

Based on the results of ROC curves analysis, seminal plasma parameters with AUC equal or greater than 0.706 were selected to perform cluster analysis. Our selection confirmed that in the case of seminal plasma: CLU, FUT3, and FUT4 concentrations may be taken into consideration as a set of additional parameters helpful to distinguish seminal OAT group of patients from men with normal seminal parameters and subjects with decreased sperm motility and abnormal morphology. On the other hand, the cluster analysis made for selected serum parameters with AUC equal to or greater than 0.710, which were CLU and FUT4 concentrations, together with relative reactivities of serum CLU glycans with UEA and LCA, enabled the determination of an additional panel of parameters helpful in differentiation of normozoospermic patients with fertility problems from those infertile with abnormal sperm morphology, reduced sperm motility, and count.

CLU concentration and the profile and degree of its fucosylation seem to be more useful for differentiating normozoospermic infertile patients from those with abnormal sperm count, motility, and morphology when the material examined is serum, not seminal plasma. The results of our research shed some light on the association between semen characteristics, which is the basis for standard semen analysis, and the expression of clusterin, both in human seminal plasma and serum, and the profile and degree of its fucosylation. In our opinion the present research is a good starting point for further investigations on the role of clusterin glycosylation in male fertility, also in the context of its role in oxidative-antioxidant balance and chaperone activity, and can be used to guide future research directions.

## Conclusions

The observed differences in concentration values of serum CLU and FUT4 as well as in the expression of core fucose and fucose α1,2-linked, being a part of Le^y^ oligosaccharide structures, in CLU glycans between the normozoospermic group of patients with fertility problems, however, and other groups examined, suggest that the disturbances in sperm count, motility and morphology are not the only cause of male fertility problems. Idiopathic infertility may result from many various changes at the molecular level, e.g., in synthesis and/or variability of glycoproteins glycosylation. This is the key to the proper fertilization process and expression of enzymes involved in the glycoproteins fucosylation. On the other hand, the lack of similarities between levels of parameters examined in blood serum and seminal plasma may suggest the differences in mechanisms of glycoproteins synthesis and those responsible for glycoproteins glycosylation. This is confirmed by observed differences in the concentration values of seminal plasma CLU, FUT3 and FUT4 observed between the OAT group and the rest of seminal plasma groups (N, T, AT), indicating that decreased sperm count may be related to these parameters expression. The lack of a representative control group of normozoospermic men with proved fertility makes it impossible to verify if the parameters analyzed in this study may be useful as biomarkers of idiopathic male infertility. This is the limitation of our study and it needs to be examined in further study. It is noteworthy that serum CLU concentration and expression of core fucose and fucose α1,2-linked in glycan structure of CLU seems to be good markers differentiating normozoospermic men from those with abnormal sperm count, motility, and morphology. Still, we cannot say the same about the seminal plasmas we studied, because despite the demonstrated weak correlations between selected semen parameters such as the total number of sperm in the ejaculate, the total number of sperm with progressive movement and the number of sperm with normal morphology versus the glyco-parameters analyzed in the study, no significant differences were found between the studied seminal plasma groups. Another important aspect of our study worth emphasizing is that the glycan-lectin reaction observed in the lectin-based ELISA method mimics the reactions that occur in male organism, showing the availability of sugar residues for endogenous ligands and thus deepening the knowledge about the mechanisms of these interactions. On the other hand, the unanswered questions may be an inspiration to undertake further research by our team and other researchers.

## Supplementary Information

Supplementary Information.

## Abbreviations

WHO: World Health Organization
CLU: Clusterin
FUT3: Fucosyltransferase 3
FUT4: Fucosyltransferase 4
LTA: *Lotus **tetragonolobus* agglutinin
UEA: *Ulex europaeus* agglutinin
LCA: *Lens culinaris* agglutinin
Le: Lewis oligosaccharide structures
Le^a/x/b/y^: Lewis^a/x/b/y^ oligosaccharide structures
ELISA: Enzyme-linked immunosorbent assay
Lectin-ELISA: ELISA test with lectins
sLe: Sialyl-Lewis oligosaccharide structures
