# Supplementary material for: The possible association of clusterin fucosylation changes with male fertility disorders
Source: Sci Rep. 2021 Aug 2;11:15674. doi: 10.1038/s41598-021-95288-w (PMC8329075; doi:10.1038/s41598-021-95288-w)
Supplement: Supplementary file 1 — Supplementary Information. [file 41598_2021_95288_MOESM1_ESM.docx]

**The possible association of clusterin fucosylation changes with male fertility disorders**

Ewa Janiszewska^1^, Izabela Kokot^1^, Iwona Gilowska^2,3^, Ricardo Faundez^4^, Ewa Maria Kratz^1*^

^1^ Department of Laboratory Diagnostics, Division of Laboratory Diagnostics, Faculty of Pharmacy, Wroclaw Medical University, Borowska Street 211A, 50-556 Wroclaw, Poland;
e-mail: ewa.janiszewska@student.umed.wroc.pl

^2^ University of Opole, Institute of Health Sciences, Collegium Salutis Humanae, Katowicka Street 68, 45-060 Opole, Poland; e-mail: iwona.gilowska@uni.opole.pl

^3^ Clinical Center of Gynecology, Obstetrics and Neonatology in Opole, Reference Center for the Diagnosis and Treatment of Infertility, Reymonta Street 8, 45-066 Opole; e-mail: igilowska@ginekologia.opole.pl

^4^ InviMed Fertility Clinics, Rakowiecka Street 36, 02-532 Warsaw, Poland; e-mail: ricardo.faundez@invimed.pl

**Supplementary Materials**

**Figure 1S.** Schematic representation of lectin-ELISA procedure. **A** - ELISA plate with goat anti-human CLU antibody capture; **B** - oxidation of oligosaccharides of anti-human clusterin polyclonal antibody with sodium meta-periodate solution (represented by ‘IO_4_^-^‘ pictogram); * this IO_4_^-^ step was performed in case of LCA reactivity detection; **C** - blocking of free binding sites using BSA blocking buffer; **D** - overnight incubation of ELISA plate with BSA blocking buffer; **E** - seminal or serum clusterin incubation (marked as ‘C’ pictogram); **F** - clusterin reduction (using dithiotreitol, DTT); **G** - fucose recognition by specific biotinylated lectins; **H** - fucose-lectin complexes incubation with ExtrAvidin (labeled with alkaline phosphatase); **I** - detection of fucose-lectin complexes using disodium para-nitrophenyl phosphate. Each incubation step was performed in standardized conditions with gentle shaking. Between each lectin-ELISA step three times washing was performed using washing buffer 10 mM TBS 0.1% Tween20, pH=7.5 (except washing after step B and F, where 10 mM TBS, pH=7.5 was used).

**Figure 2S.** Dendrogram of cluster analysis of seminal plasma samples. The cluster analysis was performed for parameters which simultaneously comply the following criteria: they allow for the differentiation of study groups and in the ROC curve analysis had moderate or high clinical value (AUC ≥0.706). Each seminal plasma sample is represented by a vector of three parameters CLU, FUT3 and FUT4.

**Figure 3S.** Dendrogram of cluster analysis of serum samples. The cluster analysis was performed for parameters which simultaneously comply the following criteria: they allow for the differentiation of study groups and in the ROC curve analysis had moderate or high clinical value (AUC ≥0.710). Each serum sample is represented by a vector of four parameters: CLU, FUT4, UEA and LCA.

| **Parameter** | **total sperm number**  (mln per ejaculate) | **sperm progressive motility**  (%) | **normal sperm morphology**  (%) |
| --- | --- | --- | --- |
| **CLU**  (ng/mL) | *r=* -0.409  *p=* 0.001 | *r=*  -0.317  *p=* 0.009 | NS |
| **FUT3**  (ng/mL) | NS | NS | *r=*  -0.300  *p=* 0.014 |
| **FUT4**  (ng/mL) | NS | NS | NS |
| **LTA**  (AU) | NS | NS | NS |
| **UEA**  (AU) | NS | NS | *r=*  0.270  *p=* 0.027 |
| **LCA**  (AU) | NS | NS | NS |

**Table 1S**. The correlations between analyzed seminal plasma parameters and selected criteria of standard semen analysis

CLU - CLU concentration; FUT3 - FUT3 concentration; FUT4 - FUT4 concentration; LTA - relative reactivity of CLU glycans with *Lotus tetragonolobus* agglutinin; UEA - relative reactivity of CLU glycans with *Ulex europaeus agglutinin*; LCA - relative reactivity of CLU glycans with *Lens culinaris* agglutinin. NS - not significant. A two-tailed p-Value of less than 0.05 was considered significant.
